# Controlling Vertical Diffusion with an Al_2_O_3_ Back Interface Layer for Stable High-Performance InZnO TFTs

**DOI:** 10.1021/acsomega.5c11257

**Published:** 2025-11-26

**Authors:** Se-Hyeong Lee, So-Young Bak, Hyeongrok Jang, Minseong Kim, Sungjae Kim, Hye-Ji Yoon, Hyeonjeong Ji, Moonsuk Yi

**Affiliations:** † Semiconductor Specialization University Support Program, 34996 Pusan National University, Busan 46241, Republic of Korea; ‡ Department of Electrical and Electronics Engineering, 34996 Pusan National University, Busan 46241, Republic of Korea

## Abstract

To improve electrical performance and bias stability for low-power applications, indium–zinc oxide (IZO) thin-film transistors (TFTs) were fabricated with an Al_2_O_3_ back interface layer that enables vertical diffusion control, together with HfO_2_/Al_2_O_3_ gate insulators. In our previous work, we achieved high switching performance in TFTs by employing high-*k* gate insulators via atomic layer deposition (ALD), but significant gate bias instability remained. To further enhance electrical performance and bias stability, vertical diffusion was precisely controlled through modulation of deposition time and oxygen partial pressure (OPP) during Al_2_O_3_ back interface layer formation using RF magnetron sputtering. As a result of controlled vertical diffusion, Al cations diffused from the Al_2_O_3_ back interface layer, significantly suppressing oxygen vacancy formation. Devices with the Al_2_O_3_ back interface layer deposited under optimized conditions exhibited enhanced electrical properties, including a saturation carrier mobility of 14.4 cm^2^/V·s and a subthreshold swing of 0.23 V/dec. Notably, under room-temperature negative bias stress, the threshold voltage shift was reduced from −1.75 to – 0.55 V, demonstrating a significant improvement over conventional IZO TFTs. We employed X-ray photoelectron spectroscopy (XPS), transmission electron microscopy (TEM), and energy-dispersive spectroscopy (EDS) to investigate the mechanisms underlying these improvements. By engineering the Al_2_O_3_ back interface layer to control vertical diffusion, this work provides a viable pathway for realizing low-power, high-performance oxide TFTs for next-generation display backplanes.

## Graphical abstract

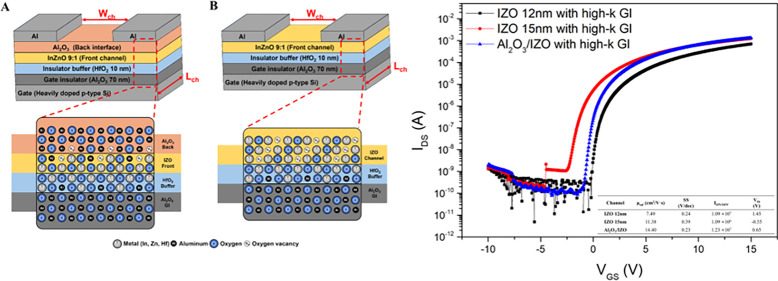

## 1 Introduction

Next-generation displays, which are attracting increasing attention in the current display market, are required to exhibit key features such as low power consumption, high resolution, high refresh rates, transparency, and mechanical flexibility.^1,2^ In emerging fields such as biomedical applications and electric vehicles (EVs), display devices demand minimal power consumption to ensure portability.^3–5^ The adoption of high refresh rates above 120 Hz substantially increases display power consumption. To address the increasing power consumption in active-matrix organic light-emitting diode (AMOLED) displays, low-temperature polycrystalline oxide (LTPO) technology was developed in 2018. This approach integrates low-temperature polycrystalline silicon (LTPS) TFTs, which offer high switching speed, with indium–gallium–zinc oxide (IGZO) TFTs, which provide low power consumption and large-area uniformity, within display backplane circuits. In conventional AMOLED displays utilizing LTPS TFTs, operating at refresh rates below 60 Hz often results in luminance degradation and flickering, even when displaying static images. In contrast, LTPO displays can operate at refresh rates as low as 1 Hz when displaying static images or text, owing to the incorporation of IGZO TFTs.^6–8^ They demonstrate enhanced power efficiency by dynamically adjusting the refresh rate in response to the displayed content. However, LTPO technology still faces several limitations, including high process complexity and low yield, both of which contribute to its elevated manufacturing costs.

To overcome the limitations of LTPO displays, it is crucial to develop amorphous oxide semiconductor (AOS) thin-film transistors (TFTs) with high electrical performance as a replacement for LTPS TFTs, thereby enabling the implementation of oxide TFT-based backplanes. Indium (In)- and zinc (Zn)-based amorphous oxide semiconductors, such as IGZO, have been extensively investigated as channel materials for TFTs owing to their high electron mobility in the amorphous phase, optical transparency in the visible spectrum, and compatibility with flexible substrates.^9–11^ The high electron mobility in the amorphous phase is primarily attributed to the significant overlap of the large, spherical s-orbitals of metal cations in the oxide semiconductor matrix. Additionally, the ionization of oxygen vacancies—native defects in AOS materials—within the thin film provides free electrons to the channel.^12,13^ However, these oxygen vacancies serve as trap sites within the channel layer and at the channel–insulator interface during TFT operation, thereby degrading the switching characteristics.^14,15^ The degradation in switching performance leads to a broader driving voltage range, which reduces power efficiency and makes the device unsuitable for low-power mobile applications.

In our previous work, we employed high-*k* dielectric thin films with an HfO_2_/Al_2_O_3_ bilayer structure as gate insulators to improve the switching characteristics of indium–zinc oxide (IZO) TFTs by achieving high gate capacitance.^16^ To obtain high-quality insulating films, the gate insulator was deposited via low-temperature atomic layer deposition (ALD) at 150 °C. To effectively suppress leakage current, an Al_2_O_3_ thin film with a wide band gap of 8.8 eV was employed as the gate insulator. However, aluminum (Al) cations, owing to their small ionic radius, readily diffuse into the channel layer and act as defects at the channel–insulator interface, thereby degrading the electrical performance.^17–19^ To suppress Al diffusion, an HfO_2_ buffer layer was deposited on top of the insulating film. As a result, the IZO TFTs with HfO_2_/Al_2_O_3_ gate insulators exhibited markedly improved switching characteristics over their SiO_2_-based counterparts. Despite the improved device stability under gate bias stress, the threshold voltage shift under negative bias stress (NBS) remained as large as – 1.75 V, which is still excessive for low-power display applications.

The instability of AOS TFTs under gate bias stress is primarily attributed to charge trapping at the dielectric/channel interface and within the gate insulator. Oxygen vacancies, which are located within the channel and at the channel–insulator interface, serve as charge trap sites.^20–22^ To improve bias stability, numerous studies have explored the incorporation of metal elements such as aluminum (Al), hafnium (Hf), and zirconium (Zr) into the channel layer as carrier suppressors. These metal elements possess low standard electrode potentials (SEP) and electronegativities, forming strong metal–oxygen bonds that suppress the formation of oxygen vacancies within the channel layer.^23–25^ Consequently, the switching characteristics and bias stability of AOS TFTs were enhanced by reducing the concentration of oxygen vacancies in the channel layer.

In addition, the surface of AOS materials readily interacts with ambient molecules, such as H_2_O and O_2_, which contributes to the degradation of bias stability in AOS TFTs. Among the surface-adsorbed species, water vapor (H_2_O) acts as a donor-like defect, providing free electrons to the channel and significantly contributing to the threshold voltage shift under negative bias stress (NBS) conditions.^26,27^ When H_2_O molecules are initially physisorbed onto the AOS surface, they remain electrically neutral. Under external stimuli, such as illumination or electric field, H_2_O molecules can become activated, contributing to the generation of charge carriers in the AOS layer. The photogenerated holes are trapped by deep-level defects within the channel and gate insulator, while the electrons migrate to the conduction band, where they act as free carriers.^28^ To mitigate the influence of H_2_O molecules on the channel surface, an additional passivation layer is required to effectively isolate the channel from ambient contaminants.

Therefore, in this study, we fabricated IZO TFTs incorporating Al_2_O_3_ back interface layer and HfO_2_/Al_2_O_3_ gate insulators via radio frequency (RF) magnetron sputtering and atomic layer deposition (ALD), respectively. This approach aims to enhance the electrical performance and bias stability of the devices, thereby making them suitable for low-power, next-generation display applications. Al cations in the back interface layer, acting as carrier suppressors, diffused into the IZO front channel during annealing, thereby reducing oxygen vacancies within the channel and at the channel–insulator interface. By depositing an Al_2_O_3_ back interface layer on the IZO front channel, surface reactions with H_2_O molecules were also effectively suppressed. To optimize the device performance, we systematically analyzed the electrical characteristics and stability by varying the deposition time and oxygen partial pressure (OPP) during the Al_2_O_3_ back interface layer deposition. We also investigated the chemical bonding states and the stacking structure of the deposited thin films using X-ray photoelectron spectroscopy (XPS), cross-sectional transmission electron microscopy (TEM), and energy-dispersive spectroscopy (EDS) analyses.

## 2 Materials and Methods

### Experimental Design

In this study, an Al_2_O_3_ back interface layer was applied to the IZO front channel of previously fabricated IZO TFTs with HfO_2_/Al_2_O_3_ gate insulators, with the aim of enhancing electrical performance and stability. To investigate the effect of the back interface layer, we fabricated IZO TFTs as reference devices and Al_2_O_3_/IZO TFTs as experimental devices, both incorporating HfO_2_/Al_2_O_3_ gate insulators. First, an HfO_2_/Al_2_O_3_ gate insulator (10/70 nm) was deposited onto a p-type Si substrate via low-temperature ALD. Subsequently, IZO channels for the reference devices were deposited at thicknesses of 12 and 15 nm using RF magnetron sputtering, while the IZO front channel of the experimental devices was deposited at a thickness of 15 nm. At this stage, the deposition time and oxygen partial pressure during back interface layer deposition were varied to examine their effects on the electrical performance and stability of the fabricated devices. After channel and interface layer deposition, annealing was performed as a post-treatment, followed by the deposition of source and drain electrodes via thermal evaporation. The electrical characteristics of the fabricated devices were measured using a semiconductor parameter analyzer, and their material properties were characterized by XPS, TEM and EDS analyses.

### Structure of the Fabricated TFTs

Figure 1A,B show the cross-sectional schematics of the inverted staggered Al_2_O_3_/IZO TFTs and IZO TFTs (with IZO channel thicknesses of 12 or 15 nm), which were fabricated as the experimental and reference devices, respectively, both incorporating HfO_2_/Al_2_O_3_ gate insulators. STEM (scanning transmission electron microscopy) and HR-TEM (high-resolution transmission electron microscopy) images of the fabricated experimental devices are shown in Figure 2A,B, respectively. These images depict the stacked structure of the channel and gate insulator layers, as well as the individual thicknesses of each layer. The interfaces between the layers appeared sharp and well-defined, indicating good layer uniformity and minimal interfacial diffusion. Moreover, the observed stacking sequence was consistent with the schematic structure shown in Figure 1A. The thicknesses of the deposited thin films were confirmed to be 77.4 nm for the Al_2_O_3_ insulating layer, 10.3 nm for the HfO_2_ buffer layer, 9.0 nm for the IZO front channel layer, and 6.7 nm for the Al_2_O_3_ back interface layer.

**Figure 1:**
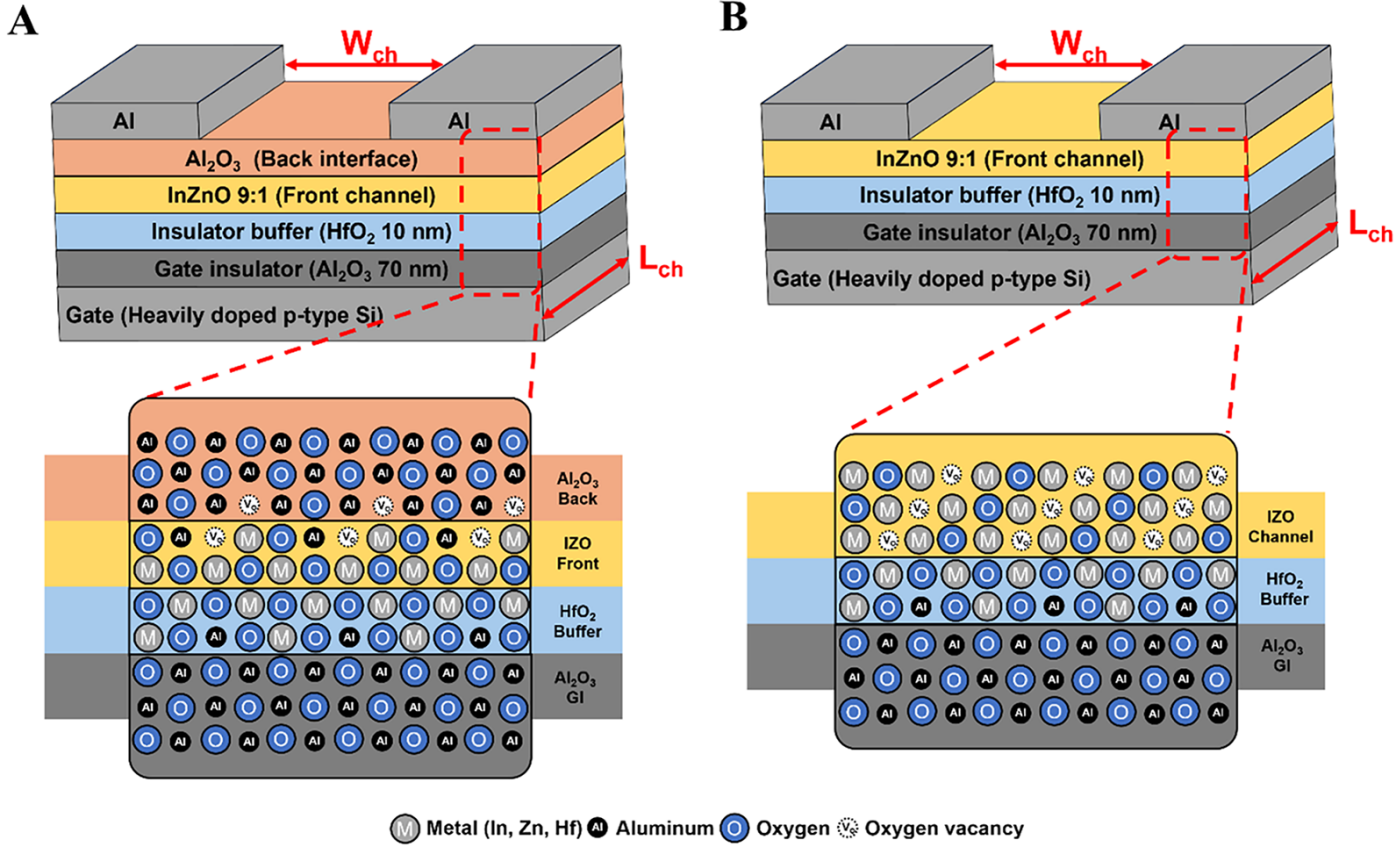
Schematic cross sections of inverted staggered-type (A) Al_2_O_3_/IZO TFTs, used as the experimental device, and (B) IZO TFTs, used as the reference device, both with HfO_2_/Al_2_O_3_ gate insulators.

### Gate Insulator Deposition

A 4-in. p-type Si wafer doped with boron (B), exhibiting a resistivity of 0.01–0.02 Ω·cm, was used as both the substrate and gate electrode. The wafer was then cleaved into 2.0 cm × 3.5 cm pieces using a diamond cutter. The high-*k* gate insulator was deposited via a low-temperature atomic layer deposition (ALD) process using a D100DC ALD system (NCD Co., Ltd.). The ALD process was carried out in a reaction chamber with an initial vacuum of 50 mTorr and a process pressure of 200 mTorr. Nitrogen (N_2_) was used as both the carrier and purge gas, and its flow rate was controlled by a mass flow controller (MFC). The Al_2_O_3_ insulating layer and HfO_2_ buffer layer were deposited at a growth temperature of 150 °C using TMA (trimethylaluminum) as the aluminum precursor, TEMAHf (tetrakis [ethyl-methylamino] hafnium) as the hafnium precursor, and H_2_O as the oxidant. The ALD cycle for Al_2_O_3_ thin-film deposition consisted of a 0.3 s TMA pulse, followed by a 20 s N_2_ purge, a 0.3 s H_2_O pulse, and another 20 s N_2_ purge. The ALD cycle for HfO_2_ thin-film deposition consisted of a 1.0 s TEMAHf pulse, followed by a 20 s N_2_ purge, a 0.2 s H_2_O pulse, and another 20 s N_2_ purge. The N_2_ purge gas flow rate was set to 60 sccm (standard cubic centimeters per minute). Cross-sectional TEM images in Figure 2 confirmed that both Al_2_O_3_ and HfO_2_ thin films exhibited a uniform growth rate of approximately 1 Å per cycle. Based on the deposition rate, the Al_2_O_3_ insulating layer and HfO_2_ buffer layer were deposited for 700 and 100 cycles, respectively. A 70 nm Al_2_O_3_ insulating layer was deposited, followed by a 10 nm HfO_2_ buffer layer to prevent the diffusion of Al cations into the channel layer.

**Figure 2:**
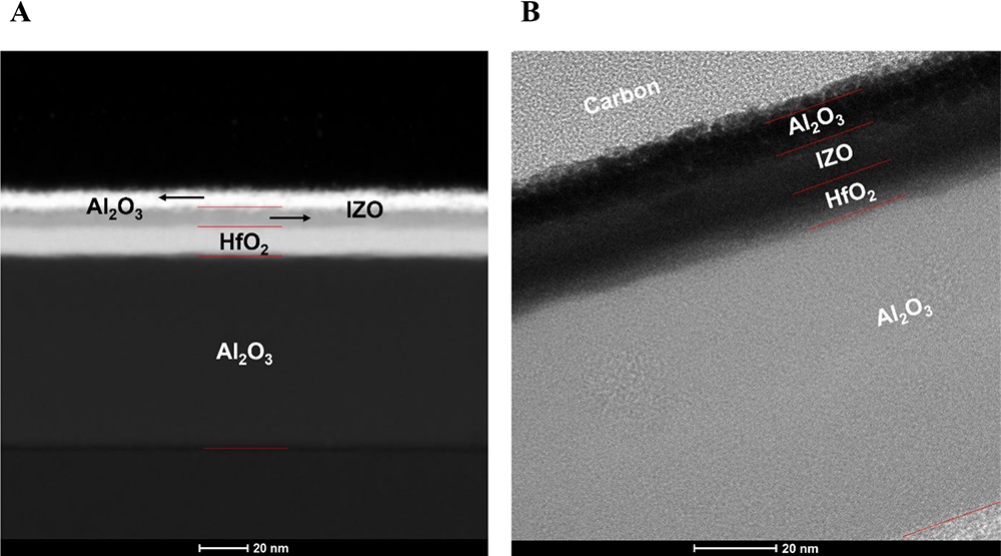
(A) Scanning transmission electron microscopy (STEM) and (B) high-resolution transmission electron microscopy (HR-TEM) images of an Al_2_O_3_/IZO TFT with HfO_2_/Al_2_O_3_ gate insulators.

### Channel and Interface Layer Deposition

The channel layer was deposited using an RF magnetron sputtering system equipped with dual targets for cosputtering. Reactive sputtering was achieved by introducing O_2_ gas together with Ar into the vacuum chamber. To prevent contamination during sputtering, the initial vacuum was set to 3.0 μTorr, and the process pressure was maintained at 2.0 mTorr. A mixed gas of Ar and O_2_ was introduced into the chamber, with the Ar flow rate fixed at 20 sccm using a mass flow controller, while the O_2_ flow rate was adjusted to control the oxygen partial pressure (OPP) in the chamber. To deposit the indium–zinc oxide (IZO) channel layer by sputtering, a 2-in.-diameter, 1/8-in.-thick single-crystal IZO target with 99.99% purity was used. The target composition was In_2_O_3_:ZnO = 90:10 (wt %). The IZO target was powered at 50 W, and the oxygen partial pressure (OPP) in the chamber during IZO channel deposition was set to 13%, corresponding to an O_2_/(Ar + O_2_) flow ratio of 2.99 sccm/(20 sccm +2.99 sccm). This condition was optimized for reactive sputtering to suppress the formation of oxygen vacancies in the channel layer.^18^ The deposition rate was approximately 1 Å/s, and the channel layer thickness was controlled by adjusting the deposition time. The channel layers of the reference IZO TFTs were deposited with thicknesses of 12 or 15 nm, while the IZO front channel layer of the experimental devices was uniformly deposited at 15 nm.

To mitigate water vapor adsorption on the IZO channel surface and to suppress the formation of oxygen vacancies via the vertical diffusion of Al cations during annealing, an Al_2_O_3_ back interface layer was deposited using RF magnetron sputtering. A 2-in.-diameter, 1/8-in.-thick single-crystal Al_2_O_3_ target with 99.99% purity was powered at 50 W, and the oxygen partial pressure (OPP) in the chamber was set to 20%, corresponding to an O_2_/(Ar + O_2_) flow ratio of 5 sccm/(20 sccm + 5 sccm). The deposition was carried out for 0, 60, 120, 240, and 300 s to investigate the effect of back interface layer deposition time on the electrical performance and stability of the fabricated TFTs. As shown in Figure 2, a 6.7 nm-thick Al_2_O_3_ back interface layer was obtained after 120 s of deposition, indicating a deposition rate of approximately 0.056 nm/s. Accordingly, the layer thicknesses corresponding to deposition times of 0, 60, 120, 240, and 300 s were estimated to be 0, 3, 7, 13, and 17 nm, respectively.

Next, while maintaining a target power of 50 W and a deposition time of 120 s, the oxygen partial pressure (OPP) in the chamber was adjusted to 13, 20, 25, and 33%. In this experiment, due to the range limitations of the O_2_ mass flow controller (MFC), the OPP was set to 25 and 33%, corresponding to O_2_/(Ar + O_2_) flow ratios of 5 sccm/(15 sccm + 5 sccm) and 5 sccm /(10 sccm + 5 sccm), respectively. Back interface layer depositions were performed under each specified condition to examine the effect of chamber oxygen partial pressure (OPP) on the electrical characteristics and stability of the fabricated devices. After the deposition of channel and interface layer, postannealing was performed on a hot plate at 250 °C for 1 h under ambient atmosphere. This annealing process was intended to facilitate atomic rearrangement within the channel layer and eliminate internal voids.^29,30^ During annealing, Al cations from the back interface layer vertically diffused into the front channel. These Al cations acted as carrier suppressors, inhibiting the formation of oxygen vacancies in the front channel.^31,32^

### Electrical Characterization

Source and drain electrodes were deposited as 100 nm aluminum (Al) thin films on the channel layer using thermal evaporation. The electrodes were patterned using a shadow mask, defining a channel width of 1000 μm and a length of 100 μm. Electrical characteristics and stability of the fabricated TFTs were measured using a semiconductor parameter analyzer (EL423, Elecs Co.). To evaluate the electrical performance, transfer characteristics were measured by sweeping the gate voltage (*V*_GS_) from – 10 to +15 V while fixing the drain voltage (*V*_DS_) at 5 V and recording the drain current (*I*_DS_). To evaluate device stability under gate bias conditions, positive bias stress (PBS) and negative bias stress (NBS) tests were performed. During the PBS and NBS tests, gate voltages of +5 and −5 V were applied for 1 h, respectively, while the drain voltage was held at 0 V. Transfer characteristics were measured at 10 min intervals to evaluate the extent of threshold voltage shift.

### Material Analysis

To analyze the chemical bonding states in the deposited thin films, X-ray photoelectron spectroscopy (XPS) and depth profiling were performed using a K-ALPHA+ system (Thermo Fisher Scientific Inc.). An Al Kα X-ray source with a photon energy of 1486.6 eV was used, and the spectra were calibrated using the C 1s peak at 284.6 eV. Depth profiling analysis revealed the distribution of Al, Hf, In, Zn, and O from the surface of the channel layer to a depth of ∼70 nm. To verify the layered structure, film thickness, and elemental distribution across each layer, transmission electron microscopy (TEM) and energy-dispersive spectroscopy (EDS) analyses were conducted using a TALOS F200X microscope (FEI) operated at 200 kV. STEM (scanning transmission electron microscopy) and HR-TEM (high-resolution transmission electron microscopy) images were acquired to confirm the layered structure and individual layer thicknesses. HAADF (high-angle annular dark-field) STEM images, along with corresponding EDS maps, were obtained to analyze the distribution of Al, Hf, In, Zn, and O across the deposited layers. In addition, EDS line scan results confirmed the elemental distribution as a function of depth, providing a detailed compositional profile across the layers.

### Parameter Extraction

The key parameters used to evaluate the electrical performance of thin-film transistors (TFTs) include carrier mobility (μ), subthreshold swing (SS), on/off current ratio (*I*_ON/OFF_), and threshold voltage (*V*_th_). These parameters were extracted from transfer characteristics, obtained by measuring the drain current (*I*_DS_) while sweeping the gate voltage (*V*_GS_) under a constant drain voltage (*V*_DS_). Carrier mobility (μ) indicates how efficiently charge carriers move through the channel layer under an applied electric field, and is expressed in units of cm^2^/V·s. As suggested by its units, carrier mobility represents the distance a charge carrier travels per unit time under a 1 V/cm electric field. Carrier mobility in thin-film transistors can be classified into two types depending on the operating region. In the linear region, carrier mobility is expressed as field-effect mobility (μ_FE_), while in the saturation region, it is defined as saturation mobility (μ_sat_), both calculated using the following eqs 33, 34. In eqs 1 and 2, *W* and *L* denote the channel width and length, respectively, while *C_i_* is the gate insulator capacitance per unit area. Field-effect mobility (μ_FE_) was calculated in the linear region using eq 1. Saturation mobility (μ_sat_) was calculated using the square of the slope of *I*_DS_^1/2^ with respect to *V*_GS_, as shown in eq 2.

$$\mu_{\mathrm{FE}}=\frac{L}{C_{i}W}\times \frac{1}{V_{\mathrm{DS}}}\times \frac{\partial I_{\mathrm{DS}}}{\partial V_{\mathrm{GS}}}$$ 1

$$\mu_{\mathrm{sat}}=\frac{2L}{C_{i}W}\times {\left(\frac{\partial \sqrt{I_{\mathrm{DS}}}}{\partial V_{\mathrm{GS}}}\right)}^{2}$$ 2

The threshold voltage (*V*_th_) is the gate voltage at which a conductive channel forms, enabling sufficient carrier accumulation at the interface between the channel layer and the gate insulator. The threshold voltage (*V*_th_) was extracted from the transfer curve by plotting the square root of the drain current (*I*_DS_^1/2^) versus gate voltage (*V*_GS_), where the extrapolated linear region intersects the axis at *I*_DS_^1/2^ = 0. A threshold voltage (*V*_th_) closer to 0 V enables more efficient power reduction during the on/off switching of the thin-film transistor.

The on/off current ratio (*I*_ON/OFF_) indicates the ratio of the drain current in the ON state to the leakage current in the OFF state of the thin-film transistor. Since AOS TFTs are widely used as switching devices, an on/off current ratio of at least 10^5^ is recommended to ensure a clear distinction between the ON and OFF states. In addition, the leakage current should remain below 1 nA to enable low-power operation. In summary, the on/off current ratio serves as an important indicator of the power efficiency of thin-film transistors. It is calculated by dividing the ON-state current by the leakage current, based on the transfer curve plotted on a logarithmic scale.^33^

The subthreshold swing (SS) is a key parameter that characterizes the switching behavior of a thin-film transistor. It is defined as the gate voltage required to change the drain current by 1 order of magnitude near the threshold voltage. The unit of SS is expressed in V/dec, and a lower value indicates faster switching, which is desirable for AOS TFTs. The subthreshold swing (SS) can be calculated using eq 3, which reflects the influence of trap density (*N_t_*) and gate capacitance (*C_i_*), and is experimentally extracted from the slope of the log-scale transfer curve.^9,34^ In eq 3, *k*_B_, *T*, and *q* denote the Boltzmann constant, absolute temperature, and charge of an electron, respectively.

$$\mathrm{SS}=\ln(10)\times \left(\frac{k_{B}T}{q}\right)\times \left(1+\frac{qN_{t}}{C_{i}}\right)=\frac{\partial V_{\mathrm{GS}}}{\partial \log(I_{\mathrm{DS}})}$$ 3

## 3 Results and Discussion

### Electrical Properties under Different Deposition Times and OPP Conditions

To evaluate the effect of back interface layer deposition on the electrical performance of the fabricated devices, a series of experiments were conducted. First, as shown in Figure 3A and Table 1, the electrical characteristics of the fabricated devices were evaluated by measuring transfer curves under varying Al_2_O_3_ back interface layer deposition times. Prior to back interface layer deposition, the IZO front channel was deposited to a thickness of 15 nm. The sputtering power of the Al_2_O_3_ target and the oxygen partial pressure (OPP) in the chamber were fixed at 50 W and 20%, respectively. The deposition time was varied from 0 to 300 s (0, 60, 120, 240, and 300 s) to control the degree of Al cation diffusion into the IZO front channel. As the deposition time increased from 0 to 120 s, the saturation carrier mobility (μ_sat_) and on/off current ratio (*I*_ON/OFF_) increased, while the subthreshold swing (SS) decreased, resulting in enhanced electrical performance of the Al_2_O_3_/IZO TFTs. These improvements are attributed to the enhanced diffusion of Al cations from the back interface layer into the IZO front channel during the postdeposition annealing process, which suppressed the formation of oxygen vacancies by acting as carrier suppressors.^31^ However, when the deposition time increased beyond 120 to 300 s, the electrical performance deteriorated, as indicated by an increase in subthreshold swing (SS) and decreases in saturation carrier mobility (μ_sat_) and the on/off current ratio (*I*_ON/OFF_). As the Al_2_O_3_ film deposited on the IZO front channel became thicker, an excessive amount of Al cations diffused into the front channel during the annealing process. Consequently, the excessive Al cations acted as defects at the channel–insulator interface and within the insulator, leading to the observed degradation in device performance.^35^

**Figure 3:**
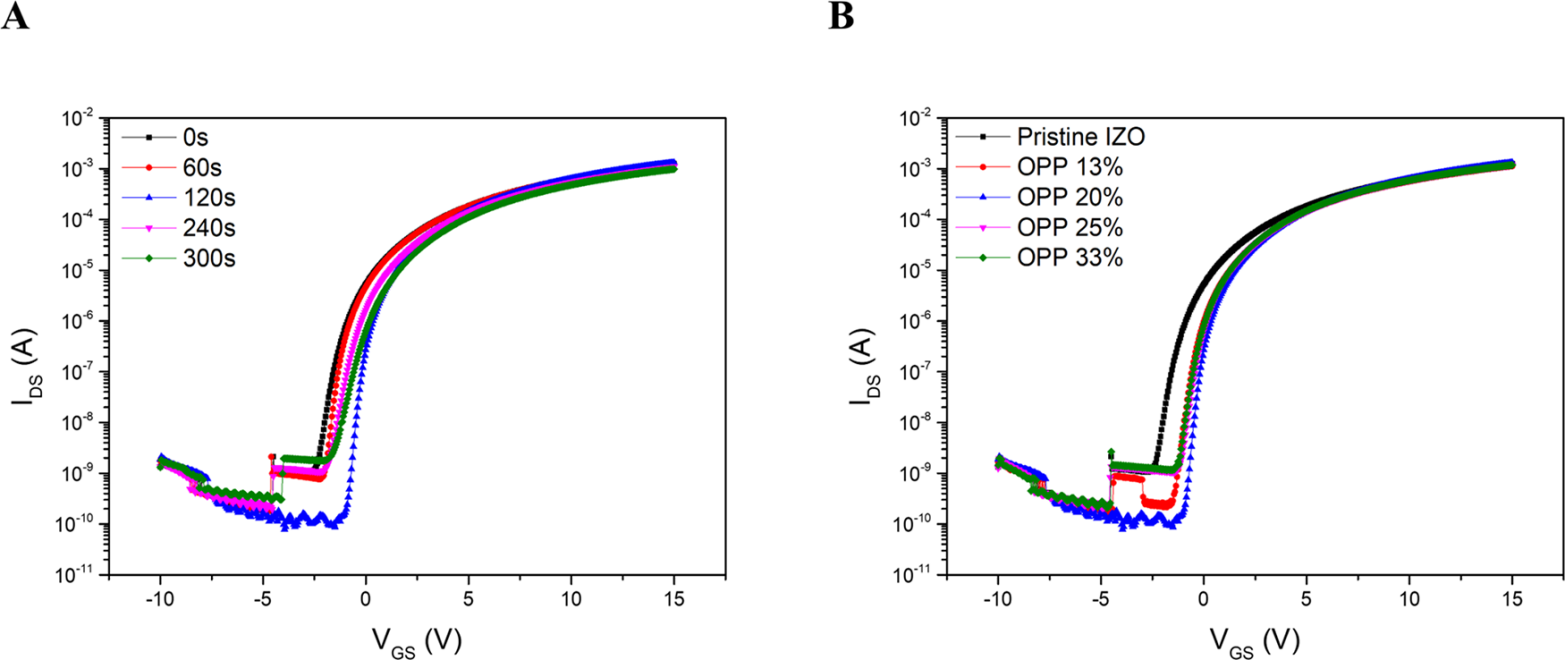
Transfer curves of Al_2_O_3_/IZO TFTs with HfO_2_/Al_2_O_3_ gate insulators, measured at varying (A) Al_2_O_3_ back interface layer deposition times and (B) oxygen partial pressures (OPP) in the chamber.

**Table 1:** Electrical Properties of Al_2_O_3_/IZO TFTs with HfO_2_/Al_2_O_3_ Gate Insulators, Evaluated with Varying Back Interface Layer Deposition Times and Oxygen Partial Pressures (OPP) in the Chamber

deposition time (s)	OPP (%)	μ_sat_ (cm^2^/V·s)	SS (V/dec)	*I*_ON/OFF_	*V*_th_ (V)
**0**	pristine IZO	11.38	0.39	1.09 × 10^6^	–0.55
**60**	**20**	11.40	0.29	1.24 × 10^6^	–0.60
**120**	**20**	14.40	0.23	1.23 × 10^7^	0.65
**240**	**20**	10.90	0.45	8.74 × 10^5^	0.05
**300**	**20**	10.85	0.56	5.31 × 10^5^	0.55
**120**	**13**	11.82	0.31	1.44 × 10^6^	0.15
**120**	**20**	14.40	0.23	1.23× 10^7^	0.65
**120**	**25**	12.94	0.35	9.04 × 10^5^	0.25
**120**	**33**	12.75	0.35	8.38 × 10^5^	0.40

Next, Figure 3B and Table 1 present the transfer curves and electrical characteristics of the devices fabricated under different OPP conditions. During the back interface layer deposition, the Al_2_O_3_ target power and deposition time were fixed at 50 W and 120 s, respectively. Simultaneously, the oxygen partial pressure (OPP) in the chamber was adjusted to 13, 20, 25, and 33%.

Compared to pristine IZO TFTs, increasing the OPP from 13 to 20% during back interface layer deposition resulted in improved saturation carrier mobility and on/off current ratio, along with a reduced subthreshold swing, indicating enhanced electrical performance. This improvement is attributed to the increased OPP during interface layer deposition, which enhanced the reactive sputtering process and effectively suppressed the formation of oxygen vacancies in the thin film. However, further increasing the OPP to 25 and 33% resulted in decreased saturation carrier mobility and on/off current ratio, along with an increased subthreshold swing. This deterioration in electrical performance is attributed to a reduced deposition rate at higher OPP conditions. When the OPP exceeded 20%, the number of argon (Ar) ions involved in the sputtering process decreased relative to the oxygen content, leading to a reduced deposition rate. The reduced deposition of Al_2_O_3_ on the front channel at higher OPP conditions led to a smaller amount of Al cation diffusion during annealing, resulting in insufficient suppression of oxygen vacancies and subsequent degradation in the electrical performance of the Al_2_O_3_/IZO TFTs.^31^ The Al_2_O_3_/IZO TFTs with HfO_2_/Al_2_O_3_ gate insulators exhibited optimal electrical performance when fabricated using a sputtering power of 50 W, a deposition time of 120 s, and an OPP of 20% during back interface layer deposition.

Figure 4 compares the transfer curves of the reference IZO TFTs and the optimized Al2O3/IZO TFTs, both fabricated with HfO2/Al2O3 gate insulators. In addition, the electrical characteristics of the IZO and Al2O3/IZO TFTs are compared and summarized in Table 2. The IZO TFTs were fabricated with channel thicknesses of 12 and 15 nm to investigate the effects of channel thickness and back interface layer deposition. Comparison of the electrical performance confirmed that increasing the channel thickness from 12 to 15 nm in IZO TFTs enhanced saturation carrier mobility, attributed to a higher carrier concentration in the channel. However, the increased channel thickness also introduced more defects within the channel and at the channel–insulator interface, leading to degradation in subthreshold swing and the on/off current ratio. Applying an Al_2_O_3_ back interface layer to the 15 nm-thick IZO front channel enabled the diffusion of Al cations, which acted as carrier suppressors, inhibited oxygen vacancy formation, and reduced defect density in the channel layer. The Al_2_O_3_ back interface layer forms a Schottky junction with the Al electrode, which increase contact resistance. However, because the deposited Al_2_O_3_ layer is very thin, under positive gate bias it allows carrier tunneling and thus exhibits Ohmic-like behavior. As a result, the contact resistance has a negligible impact on the overall device performance.^36^ Consequently, the optimized Al_2_O_3_/IZO TFTs with HfO_2_/Al_2_O_3_ gate insulators exhibited a saturation carrier mobility (μ_sat_) of 14.40 cm^2^/V·s, a subthreshold swing (SS) of 0.23 V/dec, an on/off current ratio (*I*_ON/OFF_) of 1.23 × 10^7^, and a threshold voltage (*V*_th_) of 0.65 V. The saturation carrier mobility increased by over 92% compared to the reference devices with a 12 nm-thick IZO channel and by 26% compared to those with a 15 nm-thick channel. The subthreshold swing (SS) decreased by over 41%, and the on/off current ratio (*I*_ON/OFF_) increased by more than 11-fold compared to the IZO TFTs with a 15 nm-thick channel. Table S1 summarizes the electrical characteristics of previously reported In–Zn based AOS TFTs. In general, the saturation carrier mobility is proportional to the magnitude of the gate voltage swing range. However, in this study, the devices exhibited excellent saturation carrier mobility even within a relatively small gate voltage swing range.

**Figure 4:**
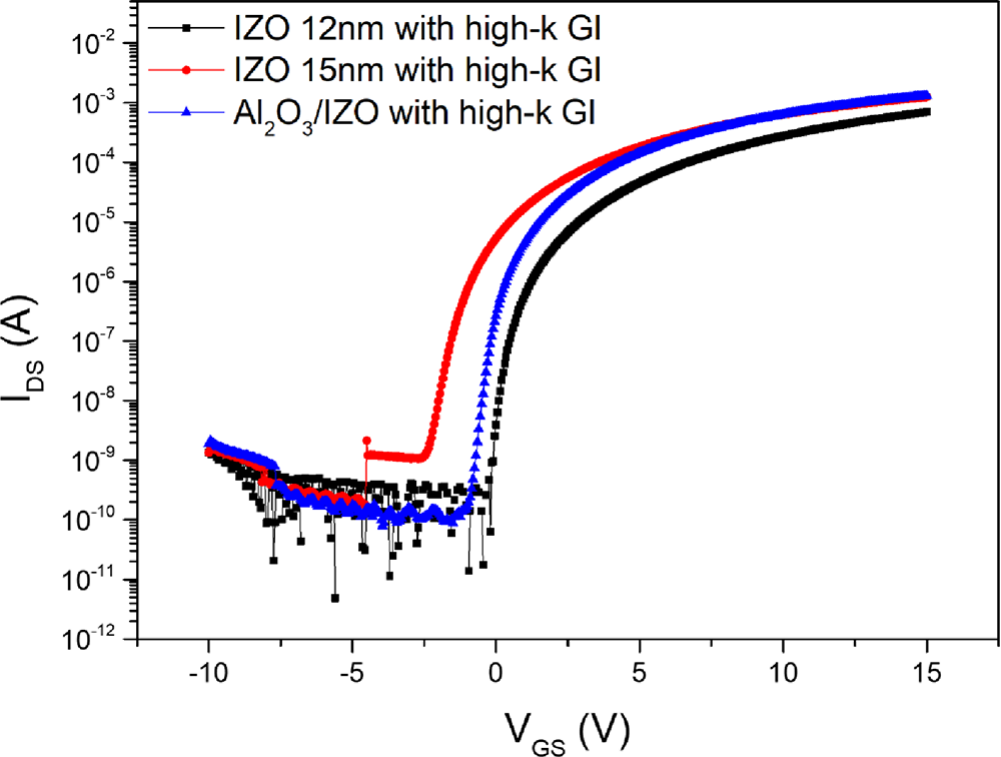
Comparison of transfer curves between experimental Al_2_O_3_/IZO TFTs and reference IZO TFTs, both employing high-*k* gate insulators (GI) with an HfO_2_/Al_2_O_3_ structure.

**Table 2:** Comparison of Electrical Properties for Experimental Al_2_O_3_/IZO TFTs and Reference IZO TFTs, Both Using High-*k* Gate Insulators (GI) based on an HfO_2_/Al_2_O_3_ Structure

channel	μ_sat_ (cm^2^/V·s)	SS (V/dec)	*I*_ON/OFF_	*V*_th_ (V)
**IZO 12 nm**	7.49	0.24	1.09 × 10^7^	1.45
**IZO 15 nm**	11.38	0.39	1.09 × 10^6^	–0.55
**Al**_**2**_ **O**_**3**_ **/IZO**	14.40	0.23	1.23 × 10^7^	0.65

### Bias Stability under Different Deposition Times and OPP Conditions

To evaluate the bias stability of the optimized Al_2_O_3_/IZO TFTs, positive bias stress (PBS, +5 V) and negative bias stress (NBS, −5 V) were applied to the gate electrode for 1 h, and transfer curves were measured at 10 min intervals. The threshold voltage shifts observed in the PBS and NBS tests, as a function of deposition time and OPP during Al_2_O_3_ back interface layer deposition, are summarized in Table 3. In the PBS test, the reference IZO TFTs exhibited a threshold voltage shift of +1.05 V, while all experimental Al_2_O_3_/IZO TFTs showed shifts of less than +1.00 V. The diffused Al cations from the back interface layer suppressed the formation of oxygen vacancies in the front channel. Consequently, the reduced defect density at the channel–insulator interface resulted in smaller threshold voltage shifts. To address the poor bias stability reported in previous research—where the threshold voltage of IZO TFTs with a 12 nm-thick channel shifted by −1.75 V under NBS conditions—we conducted further experiments. In the reference IZO TFTs, increasing the channel thickness from 12 to 15 nm reduced the threshold voltage shift under NBS conditions to −1.00 V. However, under PBS conditions, the threshold voltage shift slightly increased from +0.90 to +1.05 V. As the channel thickness increased, the density of defect states capable of trapping electron carriers at the channel–insulator interface also increased. The increased defect density deteriorated both the bias stability under PBS conditions and the electrical performance. However, the thicker channel mitigated the effect of surface reactions with H_2_O molecules—donor-like defects—leading to a reduced threshold voltage shift under NBS conditions.

**Table 3:** Threshold Voltage Shifts under Positive and Negative Bias Stress (PBS and NBS) for Al_2_O_3_/IZO TFTs with HfO_2_/Al_2_O_3_ Gate Insulators, Evaluated with Varying Al_2_O_3_ Back Interface Layer Deposition Times and Oxygen Partial Pressures (OPP) in the Chamber

deposition time (s)	deposited thickness (nm)	OPP (%)	PBS Δ*V*_th_ (V)	NBS Δ*V*_th_ (V)
0	0	pristine IZO	+1.05	–1.00
60	3	20	+0.65	–1.05
120	7	20	+0.45	–0.55
240	13	20	+0.60	–0.75
300	17	20	+0.40	–1.15
120	7	13	+0.50	–0.90
120	7	20	+0.45	–0.55
120	7	25	+0.60	–0.80
120	7	33	+0.50	–1.05

To effectively suppress surface reactions with H_2_O molecules, an Al_2_O_3_ back interface layer was deposited on the IZO front channel as a passivation layer. With the Al_2_O_3_ target power and OPP fixed at 50 W and 20%, respectively, the back interface layer was deposited for 0, 60, 120, 240, and 300 s to investigate the effect of back interface layer thickness on the bias stability of the fabricated devices. Next, the deposition time was fixed at 120 s, and the OPP was varied to 13, 20, 25, and 33% to improve device stability under NBS conditions by promoting surface reactions with O_2_ molecules, which act as acceptor-like defects. O_2_ molecules adsorbed on the AOS surface are ionized into oxygen anions (O_2_^–^) by capturing electrons from the conduction band, causing them to act as acceptor-like species.^28^ Threshold voltage shifts under NBS conditions, as a function of deposition time and OPP during back interface layer deposition, are presented in Table 3 and Figure 5. In Figure 5A, increasing the deposition time from 0 to 120 s resulted in a reduced threshold voltage shift under NBS conditions. This effect is attributed to the increased thickness of the back interface layer, which reduced the influence of H_2_O molecules on the channel surface. This overall reduction in donor-like defects contributed to the enhanced stability of the device under NBS conditions. However, extending the deposition time beyond 120 s resulted in an excessive diffusion of Al cations into the channel. These excessive Al cations acted as defect sites within the gate insulator, leading to charge injection. Consequently, the threshold voltage shift under NBS conditions increased, indicating degraded device stability.

**Figure 5:**
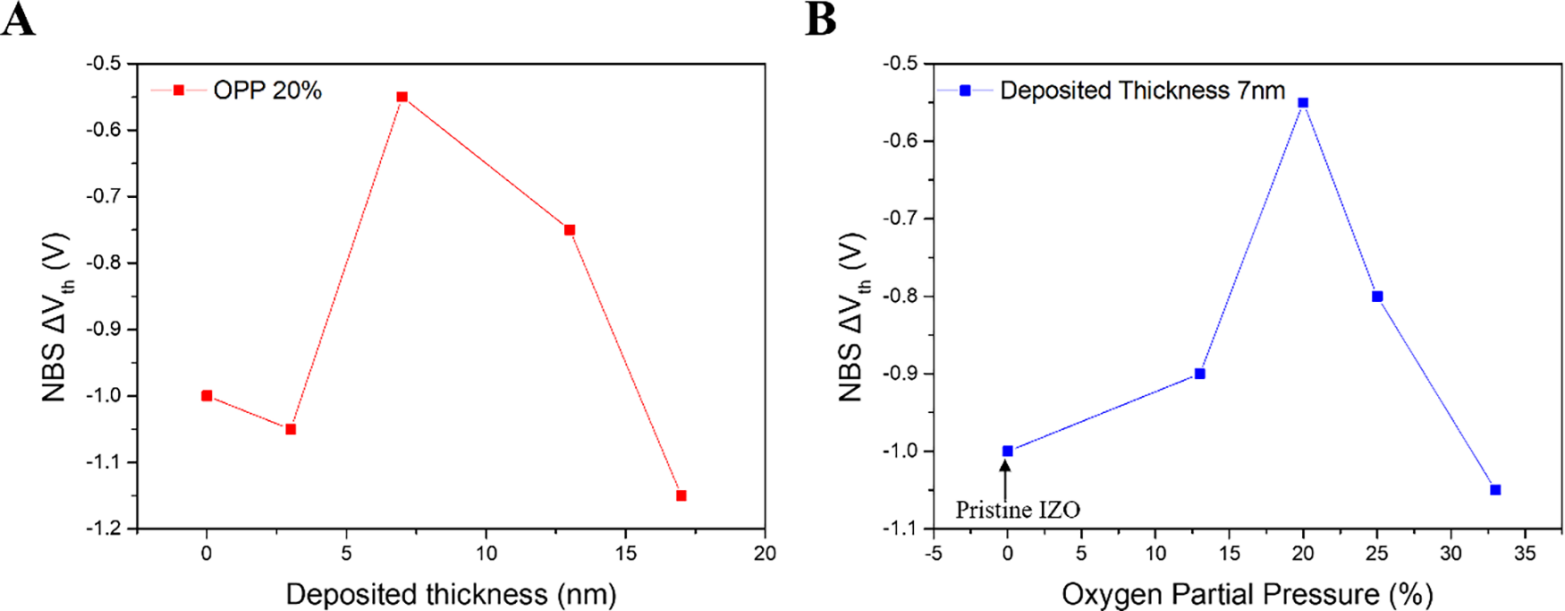
Threshold voltage shifts under negative bias stress (NBS) for Al_2_O_3_/IZO TFTs with HfO_2_/Al_2_O_3_ gate insulators, shown as a function of (A) Al_2_O_3_ back interface layer thickness and (B) oxygen partial pressure (OPP) in the chamber.

Figure 5B shows the threshold voltage shifts under NBS conditions for devices fabricated with a fixed deposition time of 120 s and varying OPP. The threshold voltage shift decreased as the OPP increased from pristine IZO TFTs (0% in Figure 5B) to 13 and 20% during back interface layer deposition. The oxygen molecules supplied to the back interface layer acted as acceptor-like species, capturing free electrons generated by H_2_O molecules. However, as the OPP increased from 20 to 33%, the number of Ar ions involved in the sputtering process became insufficient, resulting in a reduced deposition rate and fewer Al cations diffusing into the channel. As a result, the insufficient suppression of oxygen vacancy formation in the channel led to an increase in donor-like defects and a larger threshold voltage shift. Considering both the electrical performance and bias stability under gate voltage stress, the optimal deposition conditions for the Al_2_O_3_ back interface layer in Al_2_O_3_/IZO TFTs with HfO_2_/Al_2_O_3_ gate insulators were determined to be a sputtering power of 50 W, a deposition time of 120 s, and an OPP of 20%. Figure 6 graphically presents the threshold voltage shifts during PBS and NBS tests for the optimized Al_2_O_3_/IZO TFTs and pristine IZO TFTs with a 15 nm-thick channel. The optimized Al_2_O_3_/IZO TFTs demonstrated superior gate bias stability compared with the pristine IZO TFTs, showing threshold voltage shifts reduced by approximately 57% (from +1.05 to +0.45 V) under PBS and by 45% (from −1.00 to −0.55 V) under NBS.

**Figure 6:**
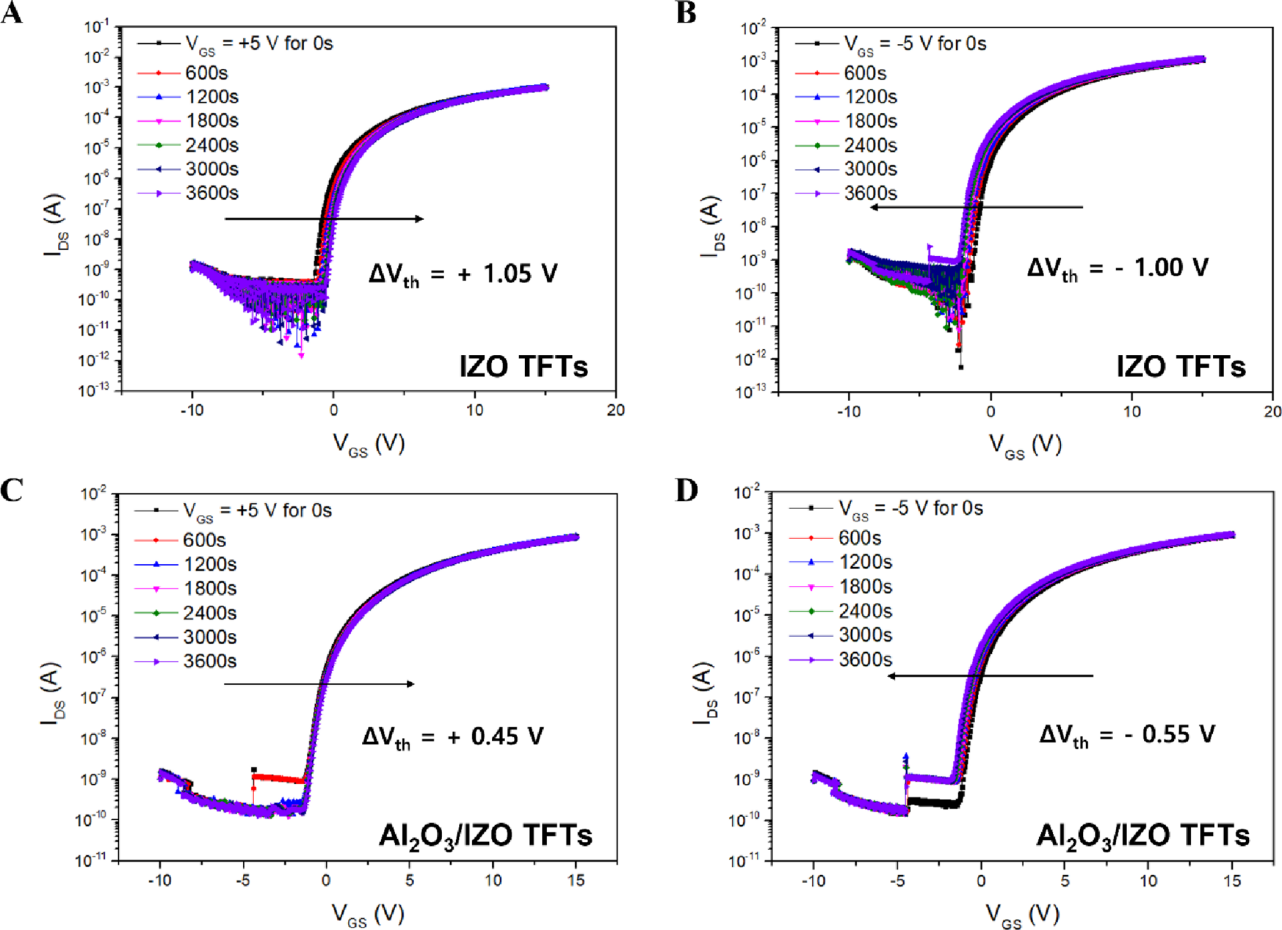
Transfer curves under gate bias stress conditions for IZO and Al_2_O_3_/IZO TFTs with HfO_2_/Al_2_O_3_ gate insulators: (A) PBS test of IZO TFTs, (B) NBS test of IZO TFTs, (C) PBS test of Al_2_O_3_/IZO TFTs, and (D) NBS test of Al_2_O_3_/IZO TFTs.

### Chemical Bonding State of the Deposited Films

#### X-ray Photoelectron Spectroscopy (XPS)

and XPS depth profiling analyses were performed to investigate how the chemical bonding structure of the Al_2_O_3_/IZO thin film deposited on an HfO_2_/Al_2_O_3_ gate insulator influences the electrical properties and stability under gate bias stress. The XPS analysis revealed the oxygen bonding states in the thin film by deconvoluting the O 1s peak into two components: a lower binding energy peak (O_L_) at 529.5 ± 0.1 eV and a higher binding energy peak (O_H_) at 531.1 ± 0.1 eV. The proportions of oxygen anions associated with ionic bonding and oxygen vacancies in the thin film were calculated as the area ratios of the O_L_ and O_H_ peaks to the total O 1s peak area (O_TOT_), respectively.^29,35^ Figure 7A,B demonstrate the deconvoluted O 1s peaks obtained from film depths of 2 nm (back interface) and 8 nm (front channel), respectively. In Figures 7A and S1, compared to previous studies on IZO TFTs with HfO_2_/Al_2_O_3_ gate insulators, the area ratio of the O_L_ peak increased from 71.02 to 82.67%, while that of the O_H_ peak decreased from 28.98 to 17.33%.^16^ In other words, compared to the IZO TFTs, the fabricated Al_2_O_3_/IZO TFTs exhibited a lower ratio of oxygen vacancies and a higher ratio of ionic oxygen anions in the back interface layer. The Al_2_O_3_ back interface layer functioned as a passivation layer, effectively suppressing the influence of H_2_O molecules on the channel surface. Additionally, the incorporation of Al cations into the channel suppressed oxygen vacancy formation, leading to a reduction in donor-like defects. In Figure 7B, the XPS results for the front channel showed that the area ratio of the O_L_ peak—corresponding to ionic metal–oxygen (M–O) bonds—increased to 88.24%. These results further confirm that an appropriate amount of Al cations was effectively introduced into the channel via vertical diffusion during the annealing process. The introduced Al cations suppressed oxygen vacancy formation, thereby enhancing the electrical performance.

**Figure 7:**
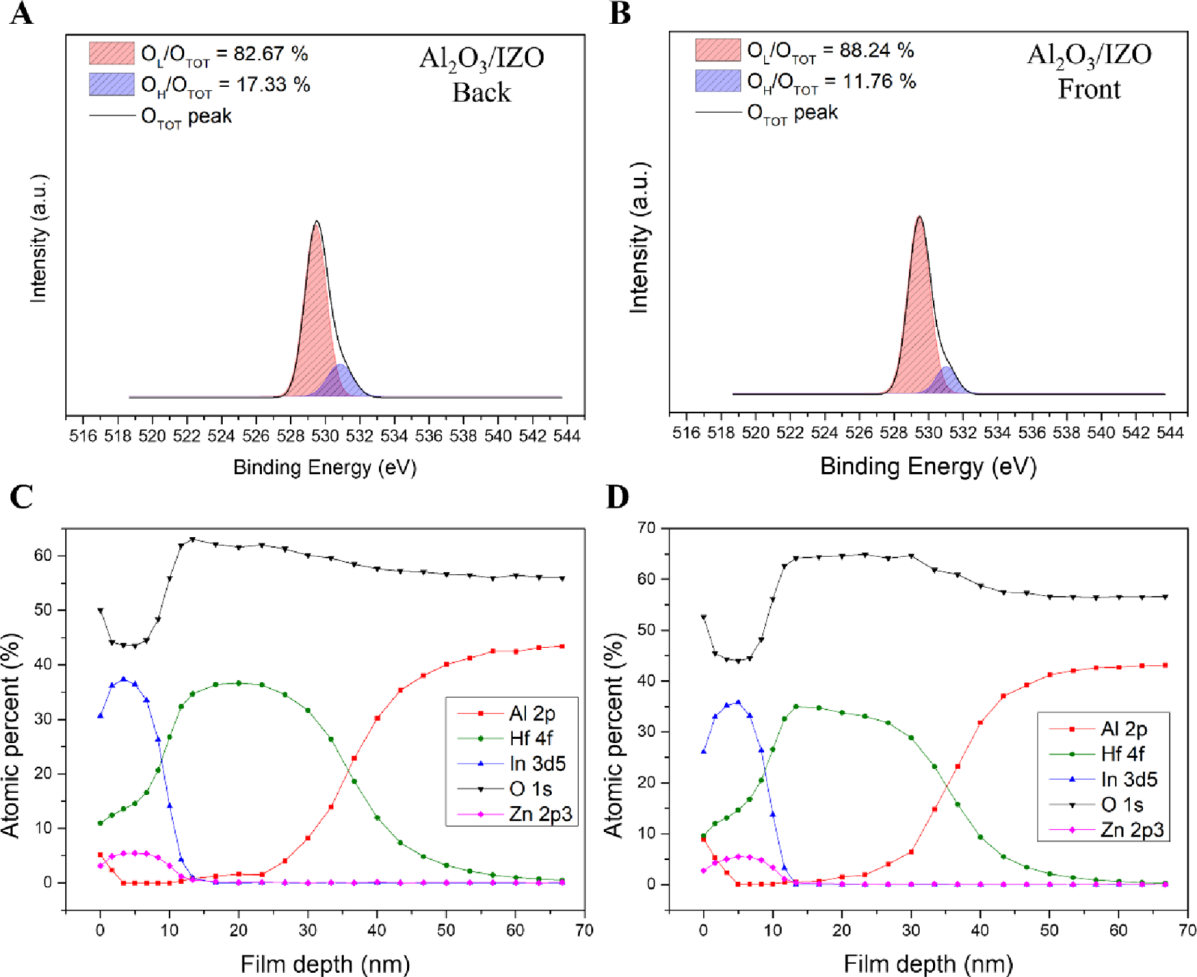
X-ray photoelectron spectroscopy (XPS) analysis of Al_2_O_3_/IZO TFTs with HfO_2_/Al_2_O_3_ gate insulators: (A, B) deconvoluted O 1s peaks at film depths of 2 nm (back interface) and 8 nm (front channel), respectively; (C, D) depth profiles for back interface layer deposition times of 120 and 240 s at 20% OPP.

#### Depth Profiling of the Deposited Films

Figure 7C,D show the XPS depth profiling results for devices with Al_2_O_3_ back interface layer deposition times of 120 and 240 s, respectively. These results revealed the extent of Al cation diffusion into the channel as a function of back interface layer deposition time. Compared to the 120 s condition, a higher concentration of Al cations was observed on the channel surface and within the channel layer in the film deposited for 240 s. The amount of Al cations diffused and injected into the channel was also higher. Additionally, based on the distribution of the Hf element, a higher concentration of Al cations was observed within the HfO_2_ buffer layer at a deposition time of 240 s. Consequently, when the back interface layer deposition time exceeded 120 s, the excessive accumulation of Al cations at the channel–insulator interface acted as defects, resulting in degraded electrical performance of the fabricated devices. These Al cations also acted as defect sites within the gate insulator, causing significant threshold voltage shifts under NBS conditions due to charge injection.

Figure 8 presents HAADF-STEM (high-angle annular dark-field scanning transmission electron microscopy) images of the Al_2_O_3_/IZO channel thin film on the HfO_2_/Al_2_O_3_ gate insulator, along with corresponding EDS (energy-dispersive spectroscopy) results showing the elemental distribution across the cross section. Figure 9 shows the elemental distribution as a function of depth based on an EDS line scan conducted on the same cross section. These figures indicate that the Hf element was densely distributed between the In and Zn in the front channel and the Al in the gate insulator. Due to their smaller ionic radius compared to In and Zn cations, Al cations more readily diffused into the channel, where they acted as defect sites and degraded the electrical performance. Because the HfO_2_ buffer layer was located between the channel and the insulating layer, it effectively suppressed the diffusion of Al cations and prevented intermixing at the channel–insulator interface. In addition, Hf cations, having an ionic radius similar to that of In and Zn, diffused in small amounts into the channel during the annealing process. These Hf cations also acted as carrier suppressors, inhibiting oxygen vacancy formation within the channel. As a result, the application of the HfO_2_ buffer layer effectively blocked the diffusion of Al cations from the insulating layer and suppressed oxygen vacancy formation in the channel, leading to enhanced electrical performance and bias stability. These findings are further supported by the XPS depth profiling results presented earlier.

**Figure 8:**
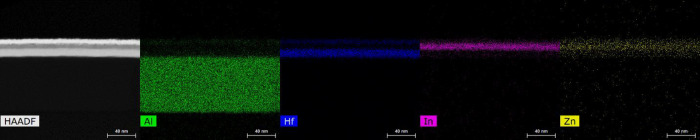
High-angle annular dark-field (HAADF) STEM images and elemental maps obtained by energy-dispersive spectroscopy (EDS) for the Al_2_O_3_/IZO channel on an HfO_2_/Al_2_O_3_ gate insulator.

**Figure 9:**
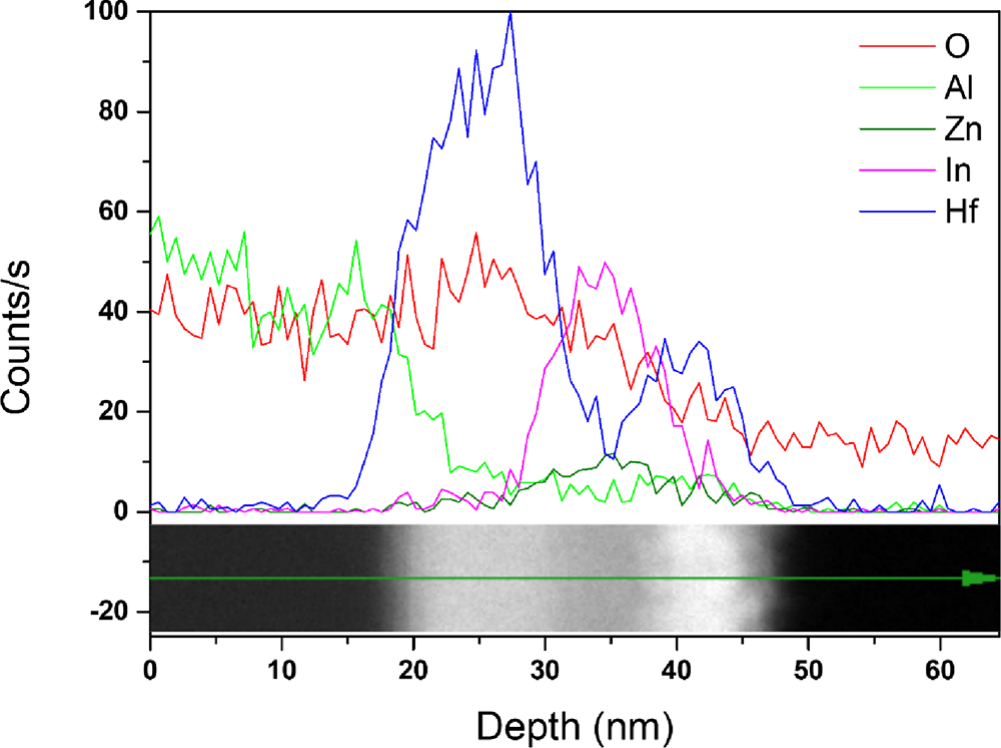
Energy-dispersive spectroscopy (EDS) line scan profile across the Al_2_O_3_/IZO channel on an HfO_2_/Al_2_O_3_ gate insulator.

## 4 Conclusions

IZO TFTs with an Al_2_O_3_ back interface layer and HfO_2_/Al_2_O_3_ gate insulators were fabricated via RF magnetron sputtering and ALD, achieving simultaneous improvements in electrical performance and stability compared with conventional IZO TFTs. By controlling the vertical diffusion of Al cations through tuning the deposition conditions of the Al_2_O_3_ back interface layer, the generation of oxygen vacancies within the channel layer was suppressed, while the influence of H_2_O molecules on the channel surface was also mitigated.

When the deposition time of the back interface layer was increased from 0 to 120 s, the saturation carrier mobility and the on/off current ratio increased, while the subthreshold swing decreased. The threshold voltage shift under NBS conditions was also reduced. These improvements are attributed to the increased thickness of the Al_2_O_3_ back interface layer, which promoted greater diffusion of Al cations into the front channel. Because of the strong affinity of Al cations for oxygen anions, the formation of oxygen vacancies was effectively suppressed. The thicker layer also mitigated the influence of surface H_2_O molecules. However, when the deposition time exceeded 120 s, the saturation carrier mobility and the on/off current ratio decreased, while the subthreshold swing increased. Furthermore, the threshold voltage shift under NBS conditions increased. Excessive Al cations acted as charge trapping defects at the channel–insulator interface and within the gate insulator, leading to deteriorated electrical performance and stability.

Increasing the oxygen partial pressure (OPP) during back interface layer deposition enhanced electrical performance and stability, with optimal characteristics observed at an OPP of 20%. Conversely, when the OPP exceeded 20%, both electrical performance and stability gradually deteriorated. These results indicate that increasing the OPP reduced the number of oxygen vacancies in the channel due to enhanced reactive sputtering during back interface layer deposition. Oxygen molecules, acting as acceptor-like species, captured free electrons generated by H_2_O molecules. However, under OPP conditions exceeding 20%, the number of Ar ions participating in the sputtering process decreased significantly, thereby reducing the amount of Al cations deposited in the back interface layer.

Based on the experimental results, the optimal device was fabricated with a back interface layer deposition time of 120 s and an OPP of 20%. Compared with the conventional IZO TFT, the optimized device exhibited a 41% reduction in subthreshold swing (SS) to 0.23 V/dec and an increase of more than 26% in saturation carrier mobility, reaching 14.40 cm^2^/V·s. It also demonstrated significantly improved bias stability, with threshold voltage shifts reduced from +1.05 to +0.45 V under PBS conditions and from −1.00 to −0.55 V under NBS conditions.

Structural and compositional analyses (XPS, TEM, and EDS) confirmed that the optimized back interface layer effectively controlled Al diffusion, validating the proposed mechanism. As a result, by adjusting the deposition conditions of the Al_2_O_3_ back interface layer and employing a high-*k* gate insulator, the vertical diffusion of Al cations was precisely regulated. This approach enables the realization of AOS TFTs for next-generation displays, providing excellent electrical performance and enhanced stability under gate bias stress. Although the fabricated devices exhibited outstanding electrical performance and bias stability, further improvements in carrier mobility are required to replace LTPS TFTs in high-resolution, high-refresh-rate OLED display backplanes.

## Supplementary Material



## Data Availability

All relevant data that support the findings of this study are available within the article and its Supporting Information file.

## Notes

XPS deconvolution results and the table summarizing electrical properties of previously reported In–Zn-based AOS TFTs (PDF)

## Abbreviations

TFT: thin-film transistor
AOS: amorphous oxide semiconductor
IGZO: indium–gallium–zinc oxide
IZO: indium–zinc oxide
GI: gate insulator
ALD: atomic layer deposition
RF: radio frequency
OPP: oxygen partial pressure
PBS: positive bias stress
NBS: negative bias stress
*V*_th_: threshold voltage
μ_sat_: saturation mobility
μ_FE_: field-effect mobility
SS: subthreshold swing
*I*_ON/OFF_: on/off current ratio
STEM: scanning transmission electron microscopy
HR-TEM: high-resolution transmission electron microscopy
HAADF: high-angle annular dark-field
EDS: energy-dispersive spectroscopy
XPS: X-ray photoelectron spectroscopy
*V*_GS_: gate voltage
*V*_DS_: drain voltage
MFC: mass flow controller
LTPO: low-temperature polycrystalline oxide
LTPS: low-temperature polycrystalline silicon
POLED: plastic organic light-emitting diode
