# Supplementary material for: Controlling Vertical Diffusion with an Al2O3 Back Interface Layer for Stable High-Performance InZnO TFTs
Source: ACS Omega. 2025 Nov 26;10(48):59976–87. doi: 10.1021/acsomega.5c11257 (PMC12772409; doi:10.1021/acsomega.5c11257)
Supplement: Supplementary file 1 [file ao5c11257_si_001.pdf]

Supporting Information

for the article entitled

'Controlling Vertical Diffusion with an Al_2O_3 Back Interface Layer for Stable High-Performance InZnO TFTs'

Se-Hyeong Lee,‡,†, So-Young Bak†, Hyeongrok Jang†, Minseong Kim†, Sungjae Kim†, Hye-Ji Yoon†, Hyeonjeong Ji† and Moonsuk Yi*,‡,†*

‡Semiconductor Specialization University Support Program, Pusan National University, Busan 46241, Republic of Korea.

†Department of Electrical and Electronics Engineering, Pusan National University, Busan 46241, Republic of Korea.

Corresponding Author

* E-mail: msyi@pusan.ac.kr

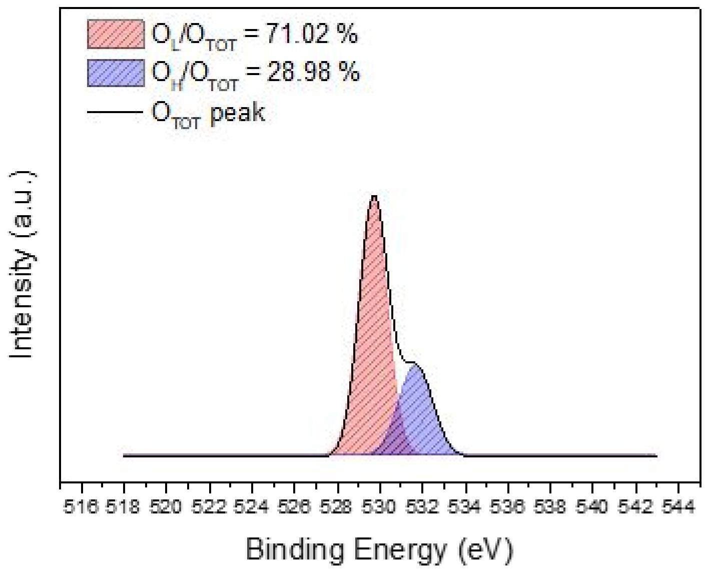

Fig. S1. Deconvolution of X-ray photoelectron spectroscopy (XPS) O 1s peak of the 10 nm-thick IZO channel layer on the HfO_2/Al_2O_3 insulator.

Table S1. Electrical characteristics of indium (In) and zinc (Zn) based amorphous oxide semiconductor (AOS) thin-film transistors (TFTs) previously reported in the literature.

Active layer	Gate insulator	V_G swing range (V)	μ_{sat} ($cm^2/V \cdot s$)	SS (V/dec)	$I_{ON/OFF}$	Ref.
IZO	SiO_2	-40 to 40	25.45	1.62	2×10^6	(1)
AIZO	SiO_2	-40 to 40	16.08	1.61	1×10^6	(1)
IGZO	SiO_2	-30 to 30	3.05	1.27	1×10^7	(2)
IGZO (UV-assisted annealing)	SiO_2	-30 to 30	15.81	0.54	1×10^7	(2)
ITZO	SiO_2	-40 to 40	17.31	0.24	3×10^7	(3)
Al_2O_3/IZO	SiO_2	-40 to 40	23.34	0.61	7×10^7	(4)
IZO	HfO_2/Al_2O_3	-15 to 10	7.49	0.24	1×10^7	(5)
Al_2O_3/IZO	HfO_2/Al_2O_3	-15 to 10	14.40	0.23	1×10^7	This work

References (Supporting Information)

1. J. Park, Y. Lim, M. Jang, S. Choi, N. Hwang, M. Yi, Improved stability of aluminum co-sputtered indium zinc oxide thin-film transistor. *Materials Research Bulletin* **2017**, *96*, 155-159.

2. Y. J. Tak, B. D. Ahn, S. P. Park, S. J. Kim, A. R. Song, K. -B. Chung, H. J. Kim, Activation of sputter-processed indium–gallium–zinc oxide films by simultaneous ultraviolet and thermal treatments. *Scientific Reports* **2016**, *6*, 21869.

3. J. Lee, J. Jin, S. Maeng, G. Choi, H. Kim, J. Kim, Enhancement of the Electrical Performance and Bias Stability of RF-Sputtered Indium Tin Zinc Oxide Thin-Film Transistors with Vertical Stoichiometric Oxygen Control. *ACS Appl. Electron. Mater.* **2022**, *4*, 1800-1806.

4. S. -H. Lee, S. -Y. Bak, M. Yi, Improved Performance and Bias Stability of Al_2O_3/IZO Thin-Film Transistors with Vertical Diffusion. *Electronics* **2022**, *11*, 2263.

5. S. -H. Lee, S. -Y. Bak, C. -Y. Park, D. Baek, M. Yi, Enhancement of electrical performance in indium-zinc oxide thin-film transistors with HfO_2/Al_2O_3 gate insulator deposited via low-temperature ALD. *Displays* **2023**, *80*, 102566.
